# Effectiveness of a Telerehabilitation-Based Exercise Program in Patients with Chronic Neck Pain—A Randomized Clinical Trial

**DOI:** 10.3390/s24248069

**Published:** 2024-12-18

**Authors:** Laura Guerra-Arencibia, Cristina Santana-Déniz, Daniel Pecos-Martín, Samuel Fernández-Carnero, Nerea de Miguel-Hernando, Alexander Achalandabaso-Ochoa, Daniel Rodríguez-Almagro

**Affiliations:** 1 Department of Nursing and Physiotherapy, University of Alcalá, 28801 Alcalá de Henares, Spaindaniel.pecos@uah.es (D.P.-M.); samuel.fernandezc@uah.es (S.F.-C.);; 2 Department of Surgery, Ophthalmology, Otorhinolaryngology and Physical Therapy, Faculty of Health Sciences, University of Valladolid, 42004 Soria, Spain; 3 Department of Health Sciences, Campus las Lagunillas, University of Jaén, 23071 Jaén, Spain; 4 Department of Nursing, Physiotherapy and Medicine, University of Almería, La Cañada de San Urbano, 04120 Almería, Spain

**Keywords:** neck pain, chronic pain, non-specific chronic neck pain, telerehabilitation, disability, exercise, manual therapy

## Abstract

Background: Non-specific chronic neck pain is a prevalent musculoskeletal disorder with a significant impact on individuals’ quality of life. The lack of consensus on effective therapeutic management complicates the establishment of standardized treatment protocols. Home exercise programs have yielded positive results. This study aimed to assess the effectiveness of a telerehabilitation program distributed through videoconferencing for patients with non-specific chronic neck pain compared to a home-based exercise program. Methods: A randomized controlled trial was conducted involving 36 participants who were divided into two groups: the experimental group (n = 18) received manual therapy combined with telerehabilitation, while the home-based group (n = 18) received the same manual therapy treatment along with recommendations for home exercises. Key outcome measures, including neck-related disability, kynesiophobia, anxiety and depression, pain intensity, pressure pain threshold, quality of life, and adherence to self-treatment, were evaluated at baseline and post-treatment. Results: No statistically significant differences were observed between groups. However, both groups demonstrated improvements in all study variables except for the mental component of quality of life immediately post-treatment. Conclusions: After eight weeks of manual therapy and exercise, both the telerehabilitation and home-based exercise programs resulted in significant improvements in disability, pain, and kynesiophobia, indicating that telerehabilitation is as effective as home-based exercise.

## 1. Introduction

Non-specific neck pain refers to discomfort in the posterior and lateral regions of the neck, located between the superior nuchal line and the spinous process of the first thoracic vertebra, without any associated signs or symptoms of major structural pathologies, specific diseases, or neurological impairments [1].

Non-specific chronic neck pain (NCNP) is the fourth leading cause of physical disability worldwide, contributing significantly to both healthcare and economic burdens [2,3]. In Spain, the annual prevalence of neck pain is 19.5% [4], with women being more affected than men, particularly in cases of NCNP (25.68% vs. 12.54%, respectively).

The heterogeneity in the characteristics of patients with neck pain [5] and the limited number of high-quality methodological studies make it challenging to standardize effective treatment protocols for this condition. Conservative interventions, such as manual therapy, therapeutic exercise, electrotherapy, and pain education, have demonstrated the most beneficial effects and are thus the most implemented in physiotherapy treatment plans [5,6]. Notably, the reviewed scientific literature indicates that multimodal approaches, combining manual therapy and exercise, yield significant and more pronounced reductions in pain [7,8] and disability [9]. These approaches also enhance patient-perceived improvements during both short-term and long-term follow-ups [10], with their effectiveness being attributed to pain modulation mechanisms [11,12].

Exercise has the most significant impact on the therapeutic management of NCNP, with structured interventions yielding superior outcomes [6,13]. Specifically, the combination of muscle strengthening, resistance, and cervical stretching exercises has proven effective in providing moderate pain relief, reducing disability, and improving strength, function, and health-related quality of life in long-term follow-ups [14,15,16]. The addition of aerobic exercise appears to further enhance these outcomes [15,17], with sustained benefits when long-term exercise habits are established [18]. Notably, exercise programs are supported by moderate-quality evidence regarding reducing the risk of recurrent neck pain episodes [19].

In summary, home exercise programs for patients with chronic pain have demonstrated promising results and are considered one of the most effective therapeutic options for reducing disability and improving health-related quality of life. However, despite the increasing number of studies on telerehabilitation, there is currently no literature supporting its effectiveness in patients with non-specific NCNP.

The aim of this study is to evaluate the effectiveness of an exercise program for individuals with NCNP by comparing two groups: one receiving the program via a web-based telerehabilitation platform and the other through in-person instruction with printed materials.

## 2. Materials and Methods

### 2.1. Study Design

A randomized, controlled, simple blind study was conducted in accordance with the CONSORT guidelines [20]. The study received approval from the Ethics Committee for Animal Research and Experimentation at Alcalá de Henares University (CEIM/HU/2020/50) and adhered to the ethical principles outlined in the Helsinki Declaration. Furthermore, this study was registered on clinicaltrials.gov (NCT04841642).

### 2.2. Subjects

The research was carried out between October 2021 and May 2022 utilizing social media and posters for participant recruitment. Prior to their inclusion in the study, all individuals were provided with comprehensive information about the research, and written informed consent was obtained from each participant.

Subjects needed to have experienced neck pain for a duration of at least three months and be between 18 and 65 years of age. Additionally, they were required to possess access to the internet and demonstrate proficiency in its management, as well as fluency in both reading and speaking Spanish. The signing of an informed consent form was also mandatory.

Individuals were excluded from the study if they had any of the following conditions: prior cervical trauma, previous neck surgery, osteoporosis, arthritis, cervical radiculopathy related to an externalized cervical hernia, vertigo, vertebrobasilar insufficiency, cancer, vertebral fractures, fibromyalgia, cognitive impairments, or psychiatric disorders. Furthermore, subjects who lacked access to or proficiency in internet management were also excluded.

### 2.3. Randomization

Patients who met the study’s inclusion criteria were randomly assigned to either the experimental or control group using a concealed allocation strategy. This was achieved through a table of random numbers generated by Epidat (Version 3.1; Consellería de Sanidade, Xunta de Galicia, Santiago de Compostela, Spain). Each participant was assigned to one of two parallel groups with an allocation ratio of 1:1, receiving either manual therapy combined with a home-based exercise program or manual therapy supplemented by a teletherapy exercise program. The randomization sequence was accessible only to the principal investigator, ensuring the integrity of the allocation process.

### 2.4. Intervention

A total of thirty-six subjects were randomly assigned to two groups. The intervention group (TRG) (n = 18) received treatment that included manual therapy combined with telerehabilitation, while the control group (n = 18) underwent the same manual therapy treatment along with a home-based exercise program (HG).

The intervention protocol lasted eight weeks and included both manual therapy and exercise, following established protocols from previous studies [17,21,22,23,24]. Throughout this period, both groups received identical manual therapy treatments consisting of one weekly session lasting 12 min (with 8 total manual therapy sessions and a total volume of 96 min). This session included 3 sets of 2 min cervical joint mobilization (with a one-minute rest between set) and 3 min suboccipital muscle inhibition, similar to the protocol proposed by Bernal-Utrera et al. [21].

The two groups were engaged in the same exercise program (3 days a week, 20–35 min per session). Both received guidance on performing the exercises from a physiotherapist during the weekly in-person sessions. While the members of the HG were not able to seek assistance on how to perform the exercises outside of these sessions, the TRG members received guidance on performing the exercises at home through videoconferencing via WhatsApp. Furthermore, the exercise performance of TRG subjects was monitored weekly through videoconferencing. If subjects encountered difficulties or had questions about the exercises, they could contact the professionals via text message for clarification. The same physiotherapist provided instructions to both groups.

The exercise program (see Supplementary Material I) was designed as a combination of the following components:

- Cervical mobility exercises.
- Isometric strengthening exercises for the cervical region.
- Neck-stretching exercises.
- Exercises targeting the strengthening of the deep flexor and extensor muscles of the neck.
- Dynamic exercises focused on the scapular girdle and shoulder.

The exercises were designed to be progressive, incorporating gradual load increases of 10% each week, as detailed in Supplementary Material II. Subjects received instructions on loading and dosage guidelines, emphasizing the importance of listening to their symptoms. Specifically, they were advised that the intensity of the exercises should not exceed a mild level of discomfort or a pain rating of 1–2 on a scale of 10.

### 2.5. Measurements

Outcome measurements were conducted on the first day of treatment and at the conclusion of the eight-week intervention period. Before the intervention commenced, demographic variables—including age, sex, height, weight, marital status, profession, and physical activity—were assessed and documented through an interview.

#### 2.5.1. Neck Pain-Related Disability

The primary outcome variable of the study was disability, which was assessed using the Spanish version of the Neck Disability Index (NDI) questionnaire. The NDI demonstrated high reliability, with an Intraclass Correlation Coefficient (ICC) of 0.98 [25].

#### 2.5.2. Quality of Life

Chronic neck pain significantly impacts patients’ quality of life, which was evaluated using the Spanish version of the Short Form-12 Health Survey (SF-12). The SF-12 demonstrated good reliability, with an ICC ranging from 0.94 to 0.96 [26].

#### 2.5.3. Anxiety

The Spanish version of the Hospital Anxiety and Depression Scale (HADS) was employed to assess levels of anxiety, exhibiting an ICC of 0.85 [27].

#### 2.5.4. Kynesiophobia

The Tampa Scale of Kynesiophobia (TSK) was used to assess kynesiophobia, with an ICC of 0.81 being demonstrated [28].

#### 2.5.5. Pain Intensity

Subjects in the study indicated the intensity of their pain using a 100 mm Visual Analog Scale (VAS), which exhibited a high level of reliability with an ICC ranging from 0.96 to 0.98 [29].

#### 2.5.6. Pressure Pain Threshold (PPT)

Cervical PPT was assessed using a digital algometer (Wagner Force Dial, Model FDK 20, Wargner Instruments, Greenwich, CT, USA) which demonstrated a reliability of ICC of 0.91 [30]. Participants were instructed to immediately inform the evaluator when the pressure became painful, at which point the mechanical stimulus was halted. Three measurements were taken at a rate of 1 point/cm^2^/s and applied perpendicularly to the skin, with an approximately 30 s rest interval between each measurement. The average value of these three measurements was used for statistical analysis.

PPT measurements with the algometer were conducted at the following anatomical locations:

1. The suboccipital region, 2 cm lateral to the external occipital protuberance, near the superior insertion of the trapezius muscle.
2. Two centimeters above the inferior insertion of the levator scapulae, located on the superior medial border of the scapula (with the patient in a prone position).
3. The upper border of the trapezius muscle, situated midway between the midline and the lateral border of the acromion.

### 2.6. Sample Size Calculation

The sample size calculation was performed using G*Power 3.1.9.2 software (latest ver. 3.1.9.7; Heinrich Heine University Düsseldorf, Düsseldorf, Germany) [31,32], referencing a clinical trial by Gialanella et al. [33]. The primary outcome variable was disability, with an effect size of d = 1.1 for disability observed in the intervention group compared to the control group. Based on an alpha level of 0.05, a beta value of 0.2, and equal group sizes (N1 = N2), the minimum required sample size was determined to be 30 subjects, with 15 per group. Accounting for an anticipated dropout rate of approximately 20%, a total of 36 subjects were included in the study. A two-tailed hypothesis test was conducted using a Student’s *t*-test to assess the difference between two independent means (two groups).

### 2.7. Blinding

The assessor who performed the assessment was unaware of the participants’ group allocation. Similarly, the analyst responsible for interpreting the results was blinded as to whether participants were assigned to the intervention or control group (assessor blinding). However, participant blinding could not be implemented.

### 2.8. Statistical Analysis

In this study, the data were processed and analyzed using IBM SPSS Statistics version 23.0 for Windows (SPSS Inc., Chicago, IL, USA). Means and standard deviations were used to describe continuous variables, while categorical variables were summarized using frequencies and percentages. The Kolmogorov–Smirnov test and Levene’s test were employed to assess the normality and homogeneity of variance of continuous variables, respectively. Additionally, to ensure comparability between the groups, potential baseline differences in clinical and morphological characteristics were evaluated. For this purpose, a Student’s *t*-test was applied to continuous variables and the Chi-square test was used for categorical variables.

To assess differences between groups over time, a Student’s *t*-test for independent samples was applied to the change scores from pre- to post-treatment. The independent progression of each group over time was assessed using a Student’s *t*-test for paired samples. Effect sizes were estimated using Cohen’s d, calculated as the mean difference between groups divided by the pooled standard deviation [34]. Following Cohen’s guidelines, effect sizes were interpreted as negligible for values less than 0.2, small for values between 0.2 and 0.49, moderate for values between 0.5 and 0.8, and large for values greater than 0.8 [34].

## 3. Results

**Figure 1:**
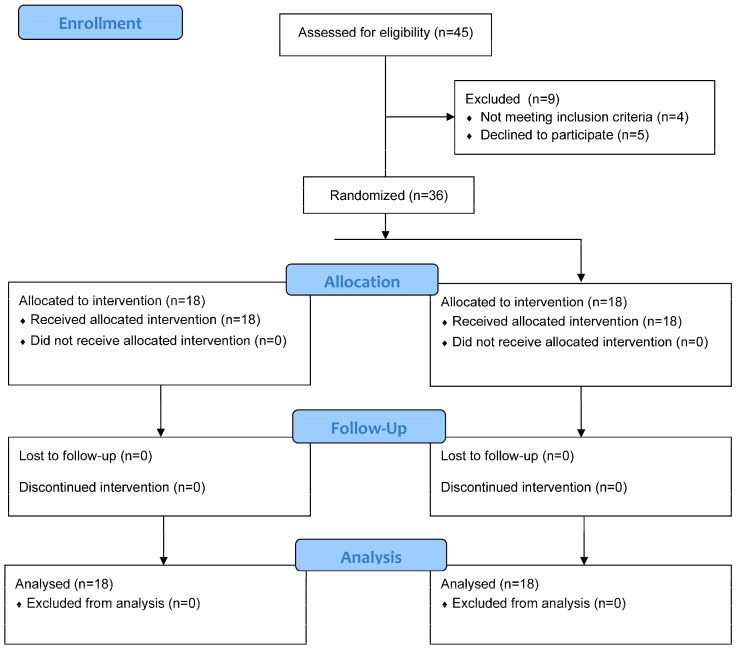
CONSORT flow diagram of the study sample.

A total of thirty-six participants met the inclusion criteria and completed the study (Figure 1). The sample had a mean age of 42.31 years (±13.68), with 63.9% being female. Baseline analysis of the morphological characteristics confirmed no significant differences between groups (Table 1). The morphological characteristics and baseline data are shown in Table 1.

The analysis conducted to assess treatment effectiveness did not yield significant differences between groups in regard to the study variables (inter-group differences *p* > 0.05), with effect sizes ranging from 0.014 to 0.409 (Table 2). However, both groups, the HG and TRG, showed immediate post-treatment improvements in all study variables except for the mental factor of quality of life (Table 2).

The HG members showed statistically significant enhancements in disability (MD: 4.72; *p* < 0.001), pain (MD: 1.24; *p* < 0.001), kynesiophobia (MD: 5.56; *p* < 0.001) and anxiety (MD: 4.06; *p* < 0.001), as well as a physical factor of SF-12 (MD: −7.26; *p* = 0.004) and pressure pain thresholds at all evaluated points, including the left suboccipital (MD: −1.28; *p* = 0.002), right suboccipital (MD: −1.06; *p* = 0.006), left levator scapulae (MD: −1.61; *p* < 0.009), right levator scapulae (MD: −1.44; *p* = 0.009), left trapezius (MD: −1.83; *p* = 0.003), and right trapezius (MD: 1.39; *p* = 0.003) (Table 2).

Similarly, the TRG members achieved statistically significant improvements in disability (MD: 4.67; *p* < 0.001), pain (MD: 1.53; *p* = 0.001), kynesiophobia (MD: 4.67; *p* = 0.002), anxiety (MD: 3.78; *p* = 0.004), and the physical component of the SF-12 (MD: −5.28; *p* = 0.009). Although no statistically significant differences were observed between groups, the TRG exhibited a greater reduction in sensitization, as measured by pressure pain thresholds, compared to the HG at most evaluated points. These included the left suboccipital (MD: −1.89; *p* < 0.001), right suboccipital (MD: −1.44; *p* = 0.001), left levator scapulae (MD: −2.33; *p* = 0.001), right levator scapulae (MD: −1.89; *p* = 0.004), and right trapezius (MD: −1.72; *p* < 0.001). An exception was observed for the left trapezius (MD: −1.44; *p* = 0.005), where improvement was also noted but was greater in the HG.

## 4. Discussion

The aim of this study was to evaluate the effectiveness of a telerehabilitation exercise program compared with a home-base exercise program for patients with NCNP. Both groups showed improvement after treatment, with no differences being observed between them, indicating that the TRG is as effectiveness as the HG. Therefore, telerehabilitation could be used in the treatment of patients with NCNP to improve participation and satisfaction [35].

The integration of therapeutic exercise into the management of chronic cervical pain has been demonstrated to be a safe and effective approach in recent years, with favorable outcomes. A number of studies have indicated that fear of movement, catastrophizing, and psychosocial factors such as anxiety and depression are significant contributors to the high levels of disability experienced by individuals with chronic neck pain. The promotion of an active approach to treatment through exercise therapy has been demonstrated to effectively reduce these factors, thereby rendering it a treatment strategy worthy of consideration [21,36]. These findings are in accordance with the results of the current study, which demonstrated that both groups exhibited improvements in these psychosocial parameters following treatment, with no significant differences being seen between them.

The findings of our study indicate a comparable statistically significant reduction in disability in both groups. Similar findings were reported by Peterson G and Peolsson A [37] in a group of patients with chronic Whiplash who participated in a neck-specific exercise program supervised by a physiotherapist with and without internet support. Nevertheless, a recent Systematic review with a meta-analysis [38] concluded that telerehabilitation is superior to other interventions in terms of improving disability in NCNP. It is hypothesized that performing these exercises may result in a perceived improvement in movement capacity in patients, potentially due to gradual exposure to load and enhanced muscle tone.

Chronic pain is defined as an unpleasant sensory and emotional experience that has the potential to significantly impact both familial and professional lives. As with other forms of chronic pain, neck pain can cause psychological distress, thereby rendering daily activities challenging and perpetuating the condition [39]. Our results demonstrated a notable pain intensity reduction in both groups. These results are consistent with those reported by Özden F. et al. [35], who demonstrated similar reductions in pain intensity in an 8-week video-based and paper-based exercise program. Nevertheless, Özel M and Kaya Ciddi [40] showed a pain decrease in a 4-week remotely supervised exercise program compared to the same 4-week unsupervised exercise program.

The reduction in pain observed in our study is likely mediated by the activation of deep cervical muscles targeted in the designed program, as studies have shown a significant relationship between the activity level of superficial neck flexor muscles and both pain intensity [41] and higher levels of pain [42]. Furthermore, the combination of manual therapy and exercise has been shown to produce better outcomes in terms of pain relief, function, patient satisfaction, and overall health compared to the application of either therapy alone in patients with chronic neck pain [1].

It is also important to consider the relationship between physical components and psychosocial factors (kynesiophobia, catastrophizing, and anxiety) that can hinder therapeutic management and adherence to treatment given that this study focuses on an exercise program for chronic cervical pain. The post-treatment results of this study demonstrate a reduction in kynesiophobia and anxiety, with no statistically significant differences being seen between the groups. These psychosocial factors directly contribute to the perpetuation of chronic pain and functional disability [43]. The ability of the cervical musculature to generate force is influenced by fear of movement; thus, gradual exposure and cervical motor control exercises help reduce kynesiophobia and associated psychosocial factors, as evidenced in the reviewed scientific literature [43,44]. Our results showed a significant reduction in these variables for both groups. These results are in consonance with those of Özden F. et al. [35], who showed improvements in kynesiophobia in both groups; however, the TRG had greater improvements.

In terms of PPT variables, no significant differences were found between groups, but both demonstrated a similarly significant increase in pressure tolerance. In this context, Bernal-Utrera C. et al. [21] reported statistically significant improvements in PPT for the group receiving manual therapy, whereas the group performing therapeutic exercise alone did not achieve statistical significance.

The decrease or loss of quality of life associated with chronic cervical pain has been extensively studied in recent years, showing a direct relationship: the greater the pain intensity, the lower the perceived quality of life [13]. Özden F. et al. [35] found improvements in the quality of life for subjects with chronic neck pain in both the video-based and paper-based groups. Furthermore, similar results were obtained in patients with chronic Whiplash [37]. Similarly, our results demonstrated a statistically significant reduction in the physical component of quality of life in both the TRG and HG, with similar improvements being observed in both. However, no improvement was found in the mental component of this variable in either group. This might indicate the need for a longer follow-up period or a more targeted treatment modality, such as pain education, as suggested by Ris I. et al. [45].

Adherence to treatment represents a key element of any physiotherapeutic intervention. The findings of this study indicate that both groups demonstrated comparable levels of adherence to the intervention plan, performing the prescribed exercises at least three days per week over the eight-week treatment period whether through telerehabilitation or a home-based program. Several factors contribute to proper adherence to home exercise programs, including monitoring (in this case, assessed through a record booklet), the complexity of the exercises, the time required for execution, the provision of feedback, and the clinical effects of the training [46].

The present study has several limitations. Firstly, the lack of long-term follow-up prevents the evaluation of the sustainability of the results obtained, indicating the need for additional research to verify the maintenance of the observed benefits. Secondly, the short evaluation period (eight weeks) and the small sample size provide limited useful information. Although eight weeks is a small period, a recent meta-analysis [36] noted that the average duration of this intervention lasts about eight weeks. Finally, it was not possible to blind the participants due to the complexities involved in implementing an appropriate blinding procedure for the selected interventions.

## 5. Conclusions

In conclusion, after eight weeks of implementing a manual therapy and exercise program delivered via telerehabilitation compared to a home-based exercise program, both groups demonstrated significant improvements in terms of disability, pain, and kynesiophobia. This study indicates that a telerehabilitation exercise program is as effective as a home-based exercise program in terms of improving disability, quality of life, anxiety, kynesiophobia, and pain, as well as being better at reducing sensitization as measured through pressure pain thresholds.

## Figures and Tables

**Table 1:** Morphologic and clinical characteristics of the sample at baseline.

Categorical		All (36)		HG (18)		TRG (18)		
		F	%	F	%	F	%	*p*
Gender	Woman	23	63.9	10	55.6	13	72.2	0.298
	Man	13	36.1	8	44.4	5	27.8	
Continuous		Mean	SD	Mean	SD	Mean	SD	*p*
Age		42.31	13.68	41.11	12.94	43.50	14.66	0.608
Height		169.72	9.15	170.22	9.64	169.22	8.88	0.748
Weight		73.81	15.51	73.11	12.24	74.50	18.55	0.793
BMI		25.50	4.37	25.19	3.48	25.81	5.19	0.681
Disability		11.97	4.90	11.44	4.08	12.50	5.67	0.526
Pain		5.50	1.51	5.60	1.47	5.41	1.59	0.762
Physical factor SF-12		42.33	9.42	41.80	9.78	42.86	9.31	0.741
Mental factor SF-12		46.36	10.52	46.30	10.11	46.42	11.21	0.974
Kynesophobia		25.36	5.62	23.83	5.33	26.89	5.62	0.103
Anxiety		13.42	5.55	12.94	5.94	13.89	5.27	0.617
Treatment days (days/week)		3.17	0.97	3.16	0.93	3.16	0.93	0.993
Pressure pain threshold								
Left suboccipital		5.64	2.74	5.89	3.25	5.39	2.17	0.591
Right suboccipital		5.47	2.52	5.83	3.19	5.11	1.64	0.398
Left levator scapulae		7.19	3.11	7.67	3.27	6.72	2.95	0.369
Right levator scapulae		6.94	2.25	7.28	2.40	6.61	2.12	0.383
Left trapezius		6.17	3.18	6.61	3.50	5.72	2.87	0.410
Right trapezius		5.97	3.03	6.44	3.28	5.50	2.77	0.357

Abbreviatures. HG: home-based exercise program group; TRG: telerehabilitation group; F: frequency; %: percentage; SD: standard deviation; *p*: *p*-value; BMI: Body Mass Index.

**Table 2:** Within-group and between-group differences at post-treatment.

Variable	Group	Post-Treatment		Intra-Group Differences			Inter-Group Differences				ES
		Mean	SD	MD	SD	*p*	MD	95% CI		*p*	d
								Lower	Upper		
Disability	HG	6.72	3.83	4.72	3.61	<0.001	−0.056	−2.430	2.319	0.962	0.014
	TRG	7.83	7.15	4.67	3.40	<0.001					
Pain	HG	3.38	1.24	2.27	1.07	<0.001	−0.494	−1.542	0.553	0.344	0.316
	TRG	3.53	1.53	1.78	1.91	0.001					
Physical Factor SF-12	HG	49.06	7.52	−7.26	9.13	0.004	1.983	−3.704	7.671	0.483	0.236
	TRG	48.14	10.51	−5.28	7.59	0.009					
Mental Factor SF-12	HG	49.30	9.74	−3.00	8.00	0.130	3.070	−1.781	7.921	0.207	0.409
	TRG	46.35	10.36	0.07	6.21	0.961					
Kinesiophobia	HG	18.28	3.54	5.56	4.02	<0.001	−0.889	−4.109	2.332	0.579	0.187
	TRG	22.22	7.86	4.67	5.39	0.002					
Anxiety	HG	8.89	5.04	4.06	2.96	<0.001	−0.278	−2.988	2.432	0.836	0.07
	TRG	10.11	7.47	3.78	4.82	0.004					
Pressure Pain Threshold											
Left Suboccipital	HG	7.17	3.29	−1.28	1.49	0.002	−0.611	−1.659	0.437	0.244	−0.395
	TRG	7.28	2.63	−1.89	1.60	<0.001					
Right Suboccipital	HG	6.89	3.36	−1.06	1.43	0.006	−0.389	−1.398	0.620	0.439	−0.256
	TRG	6.56	2.09	−1.44	1.54	0.001					
Left Levator Scapulae	HG	9.28	4.11	−1.61	2.30	0.009	−0.722	−2.308	0.863	0.361	−0.308
	TRG	9.06	3.95	−2.33	2.38	0.001					
Right Levator Scapulae	HG	8.72	3.43	−1.44	2.09	0.009	−0.444	−1.960	1.071	0.555	−0.201
	TRG	8.50	3.01	−1.89	2.37	0.004					
Left Trapezius	HG	8.44	3.33	−1.83	2.26	0.003	0.389	−1.029	1.807	0.581	0.186
	TRG	7.17	2.38	−1.44	1.92	0.005					
Right Trapezius	HG	7.83	3.20	−1.39	1.69	0.003	−0.333	−1.397	0.731	0.529	−0.209
	TRG	7.22	2.51	−1.72	1.45	<0.001					

Abbreviatures. SD: standard deviation; MD: mean difference; *p*: *p*-value; TRG: telerehabilitation group; HG: home-based exercise program group; 95% CI: 95% Confident Interval; ES: effect size; d: Cohen’s d value.

## Data Availability

Data are contained within the article.

## Supplementary Materials

The following supporting information can be downloaded at: https://www.mdpi.com/article/10.3390/s24248069/s1.
