# Supplementary material for: Effectiveness of a Telerehabilitation-Based Exercise Program in Patients with Chronic Neck Pain—A Randomized Clinical Trial
Source: Sensors (Basel). 2024 Dec 18;24(24):8069. doi: 10.3390/s24248069 (PMC11679994; doi:10.3390/s24248069)
Supplement: Supplementary file 1 [file sensors-24-08069-s001.zip › sensors-3333877-supplementary.pdf]

Supplementary material I. Exercises program description.

- Cervical mobility exercises.

Were made cervical mobility exercises described by Noormohammadpour et al. [24]. Specifically, the cervical movements of flexion, extension, lateral inclinations, and rotations were carried out in a sitting position dynamically until the end of the range of motion, but without pause at the end of it, that is, without static stretching.

- Cervical isometric strengthening exercises

Were made isometric strengthening exercises described by Chung et al. [22]. Specifically, they were executed in a sitting position, with the hand offering self-resistance to the movement of flexion, extension, lateral flexion and rotation.

- Stretching exercises of the neck

They were applied based on the protocol proposed by Daher A. et al. [17] for the following muscle groups: upper trapezius, pectoralis major and suboccipital, in conjunction with the levator scapulae proposed by Noormohammadpour P. et al. [24].

- Exercises to strengthen the deep flexor and extensor muscles of the neck

They were ruled exercises 1, 5 and 6 of the exercise protocol for the deep muscles described by Bernal-Utrera C. et al. [21], in combination with those proposed by Falla et al. [23] in the second stage of their protocol, in which they work in the prone position for the deep neck extensors.

- Dynamic exercises (scapular girdle-shoulder)

The last block was based on a combination of dynamic exercises that include the scapular girdle and shoulder regions, according to the protocols of Daher A. et al. [17] and Noormohammadpour P. et al. [24]. in which the use of an elastic band was incorporated. In relation to the first mentioned protocol, the exercises of the muscle performance section were used: elevation of the shoulder in the scapular plane with an elastic band. From the second protocol, was added those proposed in the reinforcement section: an exercise with an elastic band for rhomboids and middle trapezius muscle and another in standing for serratus anterior muscle.

Supplementary material II. Exercises program

	Type of exercise	Series	Repetitions	Rest (s)	Execution time (s)
**Week nº1**	Mobility exercises	3	10	15	-
	Isometric strengthening exercises	5	-	5	5
**Week nº2**	Mobility exercises	2	10	10	-
	Isometric strengthening exercises	5	-	5	5
	Stretching exercises	1	-	-	20
**Week nº3**	Mobility exercises	1	10	-	-
	Isometric strengthening exercises	3	-	5	10
	Deep muscle strengthening (1er ex. Bernal-Utrera C. et al. [21])	3	10	• 3 between reps<br>• 10 between series	5
	Stretching exercises	1	-	-	20

	Type of exercise	Series	Repetitions	Rest (s)	Execution time (s)
**Week nº4**	Mobility exercises	1	10	-	-
	Isometric strengthening exercises	3	-	5	10
	Deep muscle strengthening (1er ex. Bernal-Utrera C. et al. [21])	3	10	• 5 between reps<br>• 10 between series	5
	Deep extensor muscle strengthening (Falla et al. [23])	2	10	• 5 between reps<br>• 10 between series	5
	Stretching exercises	1	-	-	20
**Week nº5**	Mobility exercises	1	10	-	-
	Isometric strengthening exercises	2	-	5	10
	Deep muscle strengthening (1er ex. Bernal-Utrera C. et al. [21])	2	10	• 3-5 between reps<br>• 10 between series	8-10
	Deep extensor muscle strengthening (Falla et al. [23])	2	10	• 3-5 between reps<br>• 10 between series	5
	Deep muscle strengthening (5º ex. Bernal-Utrera C. et al. [21])	2	8	• 10 between series	-
	Stretching exercises	1	-	-	20

	Type of exercise	Series	Repetitions	Rest (s)	Execution time (s)
**Week nº6**	Mobility exercises	1	8	-	-
	Isometric strengthening exercises	2	-	5	10
	Deep muscle strengthening (1er ex. Bernal-Utrera C. et al. [21])	2	10	• 3-5 between reps<br>• 10 between series	10
	Deep extensor muscle strengthening (Falla et al. [23])	2	10	• 3-5 between reps<br>• 10 between series	8-10
	Deep muscle strengthening (5º y 6º ex. Bernal-Utrera C. et al. [21])	2	8	• 10 between series	-
	Stretching exercises	1	-	-	20
**Week nº7**	Mobility exercises	1	8	-	-
	Isometric strengthening exercises	2	-	5	10
	Deep muscle strengthening (1er ex. Bernal-Utrera C. et al. [21])	2	10	• 3-5 between reps<br>• 10 between series	10
	Deep extensor muscle strengthening (Falla et al. [23])	2	10	• 3-5 between reps<br>• 10 between series	10
	Deep muscle strengthening (5º y 6º ex. Bernal-Utrera C. et al. [21])	2	8	• 10 between series	-
	Dynamic exercises	2	10	• 10 between series	
	Stretching exercises	1	-	-	20

	Type of exercise	Series	Repetitions	Rest (s)	Execution time (s)
Week nº8	Mobility exercises	1	8	-	-
	Isometric strengthening exercises	2	-	5	10
	Deep muscle strengthening (1er ex. Bernal-Utrera C. et al. [21])	2	10	• 3-5 between reps<br>• 10 between series	10
	Deep extensor muscle strengthening (Falla et al. [23])	2	10	• 3-5 between reps<br>• 10 between series	10
	Deep muscle strengthening (5º y 6º ex. Bernal-Utrera C. et al. [21])	2	10	• 10 between series	-
	Dynamic exercises	3	10	10 between series	-
	Stretching exercises	1	-	-	20

Abbreviations. Reps: Repetitions; s: seconds; ex: exercise.
